# Intermittent retinal artery occlusions as the first clinical manifestation of polycythemia vera: a case report

**DOI:** 10.1186/s12886-022-02423-w

**Published:** 2022-05-15

**Authors:** Jasmin Regensburger, Teresa Rauchegger, Lorin Loacker, Florian Falkner, Clemens Feistritzer, Barbara Teuchner

**Affiliations:** 1 Department of Ophthalmology and Optometry, Medical University Innsbruck, Anichstrasse 35, 6020 Innsbruck, Austria; 2 Central Institute for Medical and Chemical Laboratory Diagnostics, Medical University of Innsbruck, Anichstrasse 35, 6020 Innsbruck, Austria; 3 Tyrolpath Obrist Brunhuber GmbH, Hauptplatz 4, 6511 Zams, Austria; 4 Department of Internal Medicine, Medical University Innsbruck, Anichstrasse 35, 6020 Innsbruck, Austria

**Keywords:** Intermittent retinal artery occlusion, Polycythemia vera, Myeloproliferative neoplasm, JAK2-V617F mutation, Fluorescence angiography, Case report

## Abstract

**Background:**

Polycythemia vera (PV) is a myeloproliferative neoplasm with increased hemoglobin, hematocrit, platelet count and leukocytosis, resulting in increased blood viscosity. PV which is initially presenting with ocular symptoms is rare, but irreversible retinal vessel occlusions leading to the diagnosis of PV have been described in literature.

**Case presentation:**

We describe a patient with PV, initially presenting with attacks of monocular temporary loss of vision due to intermittent retinal artery occlusions of different retinal arteries. The patient was immediately treated with phlebotomy and the impaired arterial retinal perfusion could be restored without permanent retinal ischemia. We were able to document these transient arterial occlusions with fundus photography as well as fluorescein angiography. To the best of our knowledge, a case like this has never been documented before.

**Conclusion:**

This report is pertinent, in order to raise awareness among clinicians for polycythemia vera, as it can in fact be used as a differential diagnosis for patients with retinal artery occlusion. We would like to stress that early therapy might reverse the vessel complications.

**Supplementary Information:**

The online version contains supplementary material available at 10.1186/s12886-022-02423-w.

## Background

Polycythemia vera (PV) is a myeloproliferative neoplasm (MPN) characterized by an abnormally elevated production of blood cells by the bone marrow [1, 2]. Due to the resulting hyperviscosity, patients with PV are at high risk of developing thrombotic complications [1–4]. Ocular involvements include retinal artery occlusion, retinal vein occlusion, peripheral non-perfusion, retinal hemorrhages and visual field defects corresponding to cerebral non-perfusion [5–8]. These vascular occlusions usually lead to irreversible changes in the ocular blood flow [8–10]. We present a patient with PV with transient retinal arterial occlusions in various arteries, which re-perfused under therapy.

## Case presentation

A 54-year-old woman came to our department and complained about experiencing painless, intermittent monocular visual loss in the right eye. She described the visual loss as a gray shade lowering itself over her eye for several minutes. Since the previous day, these attacks of visual disturbance had been occurring multiple times every hour. Her medical history was significant for migraines with aura but she described that these symptoms were different than usual.

On presentation, best-corrected visual acuity was 20/25 in the right eye (OD) and 20/20 in the left eye (OS). Slit-lamp examination, intraocular pressures and fundoscopy were unremarkable, there was no afferent pupillary defect present. A spectral-domain optical coherence tomography (SD-OCT) of the macula showed no abnormalities. The visual field examination was unremarkable. At the time of the examination the patient was in an attack-free interval.

**Fig. 1:**
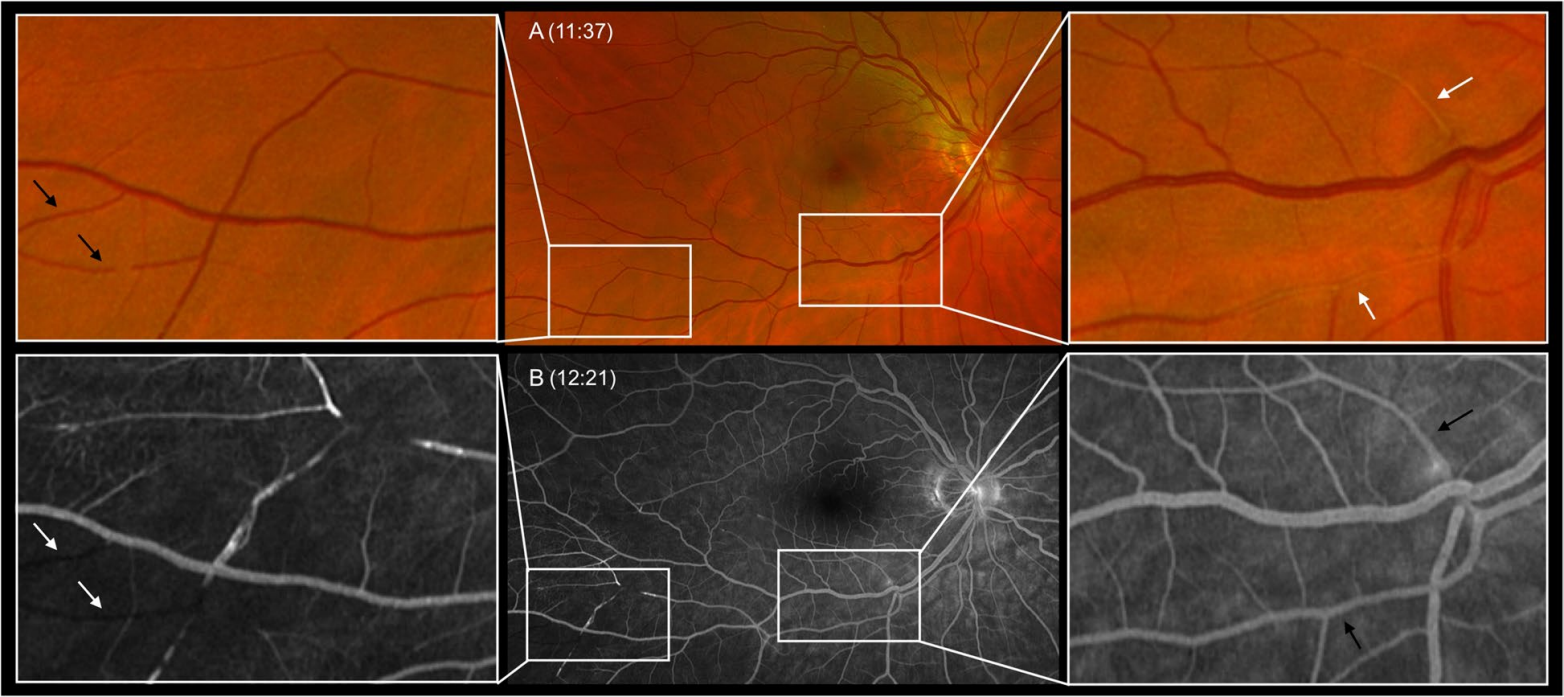
Multimodal Imaging of the right eye at presentation (**A**) Scanning laser ophthalmoscope image demonstrating small retinal artery occlusions (white arrows) (Time: 11:37), (**B**) Fluorescence angiography (FA) shows normal central blood flow (black arrows) but closed arteries in temporal periphery (white arrows) 44 min later (Time: 12:21). (White arrows indicate occluded vessels, black arrows point to vessels with perfusion)

**Fig. 2:**
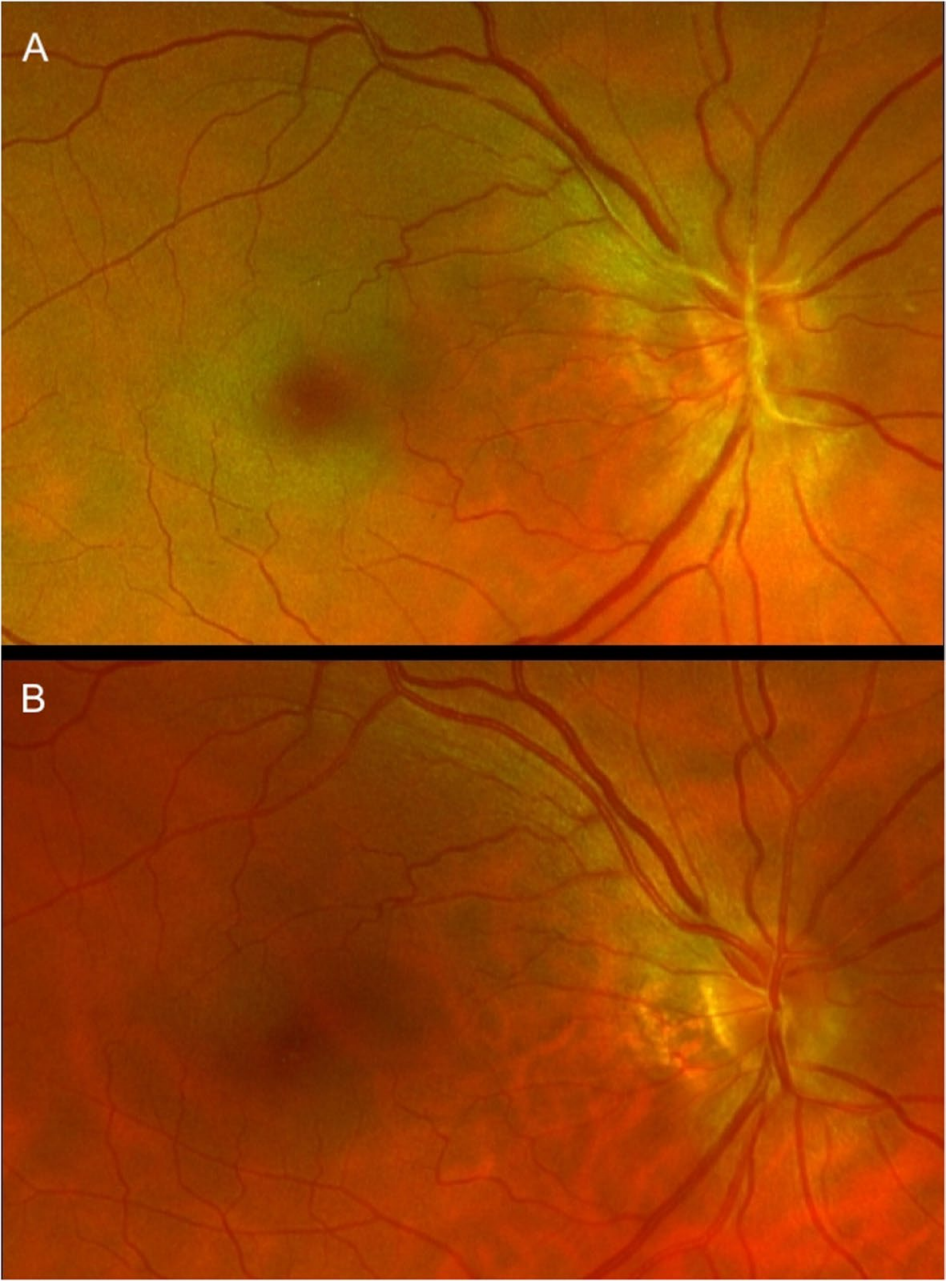
Scanning laser ophthalmoscope images of the posterior pole (**A**) One hour after initial presentation showing a central retinal artery occlusion with the initial signs of a cherry red spot (**B**) After therapy with re-perfused central retinal artery

A color-duplex sonography of the carotid arteries was implemented and showed no abnormalities. When the patient returned from the sonography, she complained about a new incident of visual disturbance. The fundus examination was repeated and at this time occlusions of two temporal retinal branches were visible (Fig. 1 A). Furthermore, the fluorescence angiography showed occlusions of inferior retinal arteries more peripheral than visible on fundoscopy and as well as an area of non-perfusion in the temporal inferior quadrant (Fig. 1 B). A re-examination 10 min later suddenly revealed a central retinal artery occlusion with a mild cherry-red spot in the macula (Fig. 2).

The immediately initiated hematological investigations were significant for the following features: leukocytosis, thrombocytosis and a polycythemia with a hemoglobin index of 200 g/l and a hematocrit of 59,2%. The diagnosis of Poliglobulia was made and the patient was immediately treated with acetylsalicylic acid 100 mg twice a day and a low molecular weight heparin. Additionally, the patient had to undergo phlebotomy (450 ml blood was taken twice). Under this treatment the hematocrit decreased from 59,2% to 44,8%.

**Fig. 3:**
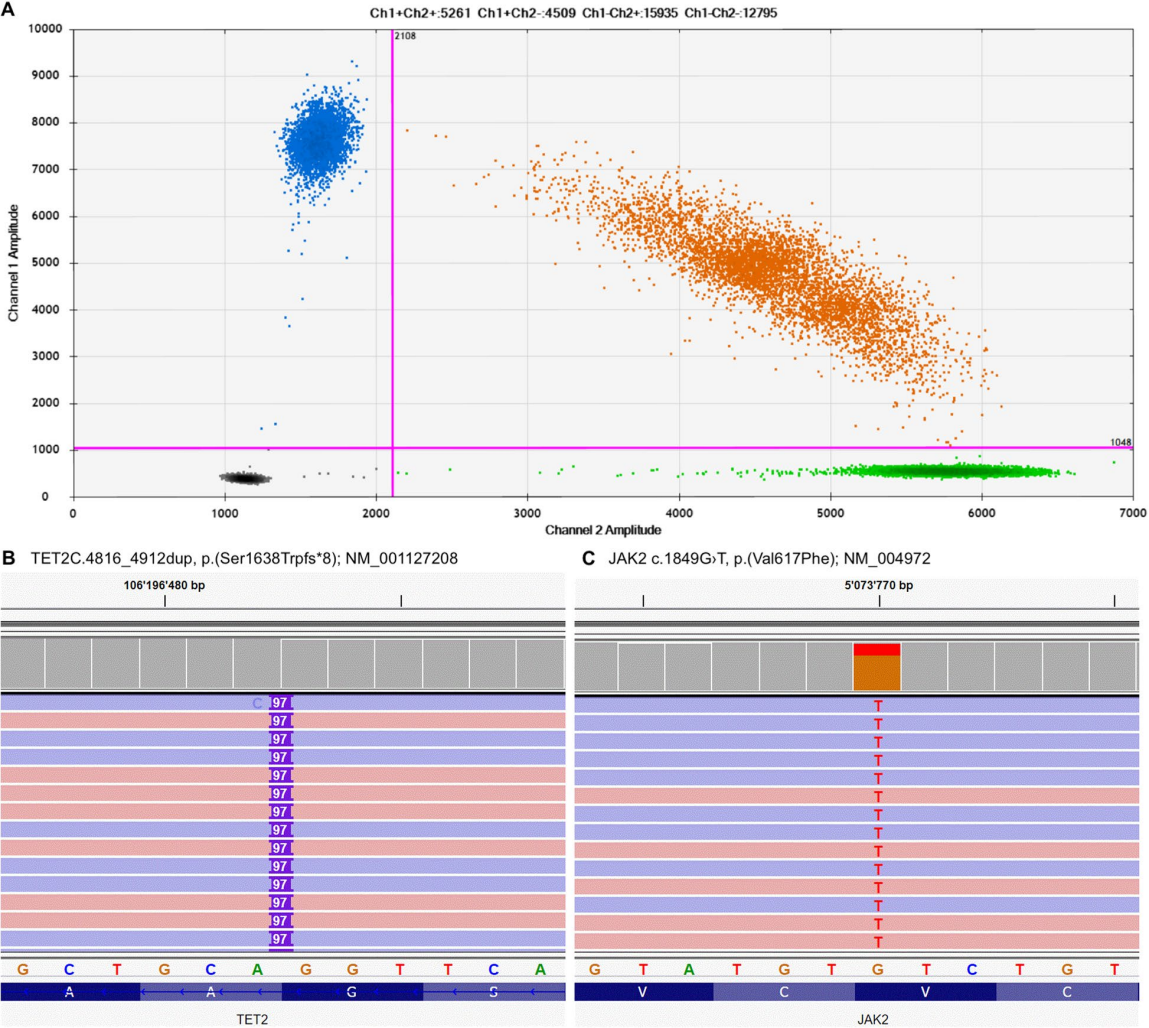
Molecular findings in peripheral blood: (**A**) digital droplet PCR 2D Scatter Plot showing the JAK2 V617F mutation (green droplets: wildtype allele, blue droplets: mutant allele, orange droplets: mixed wildtype and mutant allele). (**B**,**C**) Next-Generation-Sequencing analysis revealing a TET2 frameshift mutation and JAK2 point mutation

A PCR-screening for the most common myeloproliferative hotspot mutations (JAK2, CALR, MPL) was performed from peripheral blood leukocytes and revealed a JAK2 V617F mutation with a variant allel frequency of 28.3% by digital droplet PCR. A subsequently performed Next-generation-sequencing by a 30 genes containing myeloid panel revealed an additional TET2 frameshift mutation in exon 11 (VAF 35.8%) as it frequently occurs in later disease stages (Fig. 3).

**Fig. 4:**
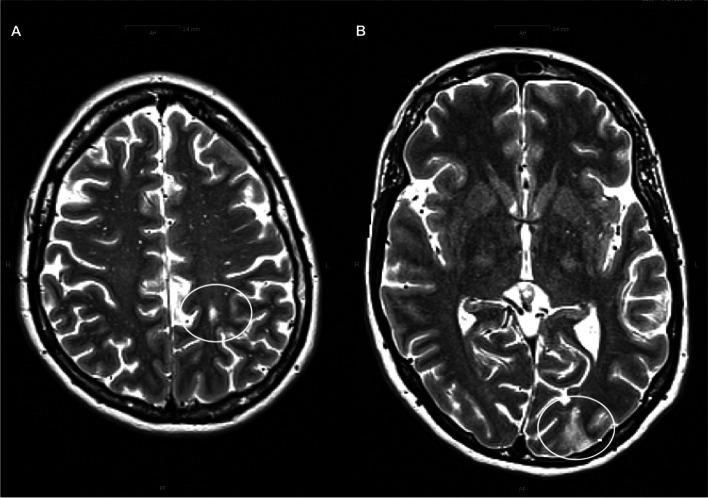
Cerebral magnetic resonance tomography demonstrated lesions in the left hemisphere from cerebral infarctions in the past (**A**) Central T2 hyperintense lesion, partly diffusion disturbed (**B**) Left occipital wedge-shaped lesion with diffusion disorder and T2 hyperintensity

A bone marrow trephine biopsy confirmed the diagnosis. It showed normocellular bone marrow with proliferated atypical megakaryopoiesis (medullary fibrosis grade 0), consistent with the picture of a myeloproliferative neoplasm type polycythemia vera (Additional Files 1, 2, 3, 4 and 5). The cerebral magnetic resonance tomography demonstrated lesions in the left hemisphere from cerebral infarctions in the past (Fig. 4).

At a follow-up ophthalmological examination, the retinal perfusion was unremarkable. On fundus examination and fluorescence angiography no retinal artery occlusions or areas of retinal non-perfusion were detectable. The patient’s visual acuity improved to 20/20 OD and she negated having ocular symptoms or incidents of temporary vision loss since the start of treatment.

## Discussion and conclusion

Polycythemia vera (PV) is a chronic clonal hematopoietic stem cell disorder characterized by an increase in the red cell mass, thrombocytosis, leukocytosis and splenomegaly and myelofibrosis [1, 2]. Mutations in the tyrosine kinase gene Janus Kinase 2 (JAK2) are the leading cause of PV. Since the elevated red blood cell mass leads to an increased hemoglobin concentration, hematocrit value and blood viscosity, patients with PV are at high risk of developing thrombosis [1–4]. In approximately 39–41% of patients, thrombosis and thromboembolic events are observed [11, 12]. In recent observational studies the most common venous events were deep vein and superficial venous thrombosis, splanchnic vein thrombosis and pulmonary embolism. Acute coronary syndrome, acute myocardial infarction, cerebrovascular arterial thrombosis and stroke were among the most common arterial events in patients with PV [13]. In our patient multiple previous cerebral infarctions were detectable.

Ocular symptoms are present in about 13,6% of the patients with PV, but PV initially also showing ocular symptoms is especially rare [14]. A few case reports described central retinal artery occlusion, isolated cilioretinal artery occlusion or central retinal vein occlusion leading to the diagnosis of PV. In these cases, the vascular occlusions led to irreversible changes in the ocular blood flow [8–10]. In contrast, we were able to document vessel occlusions that re-perfused without permanent damages.

We came to the hypothesis that we witnessed an arterial perfusion impairment with transient occlusion of the central retinal artery caused by increased blood viscosity. The patient’s symptoms occurred in response to the differences in retinal blood flow. In cooperation with the Department of Internal Medicine, instant blood tests led to an early diagnosis of PV and an immediate start of therapy. In patients with PV the reduction of the hematocrit is the most important intervention in order to decrease the risk of thrombotic events [15]. In our case, the patient was treated with phlebotomy. Under this treatment the hematocrit decreased from 59,2% to 44,8% and the impaired arterial retinal perfusion could be restored without permanent retinal ischemia. Consistent with our findings, Yang et al.discovered that the arm–choroidal filling time and the artery–venous transit time was significantly prolonged in PV patients compared to controls. While the patients did suffer from visual symptoms, there was no vessel occlusion detectable. After treatment with phlebotomy to a hematocrit below 50% and Hydroxycarbamide, the choroidal and retinal blood flow improved and the visual symptoms resolved [14]. Our case report went further than this by noticing and documenting transient retinal arterial occlusions in various arteries, which re-perfused over the course of an instantly initiated therapy.

As atypical venous thrombosis (e.g. in portal vein, spleen vein, Budd-Chiari Syndrom) can be the first manifestation for an underlying undetected myeloproliferative disease including PV, several guidelines already do recommend a screening for the JAK2 V617F mutation in these patients [16, 17]. In consideration with our patient a molecular screening for the JAK2 V617F mutation in patients with visual disturbances might also be reasonable, since other routine laboratory diagnostics such as blood count (especially in early disease stages) are not always indicating or excluding a myeloproliferative disorder in a reliable way.

It is therefore possible that PV patients with visual disturbances suffer from an unnoticed intermitted vessel occlusion at first, which could subsequently lead to permanent ischemic damage. In our case, it is conceivable that the rapid start of therapy prevented irreversible damage.

In conclusion, this case illustrates a temporary loss of vision due to a lack of retinal circulation as an initial symptom of polycythemia vera. PV is a myeloproliferative neoplasm characterized by an abnormally increased production of blood cells. This results in high blood viscosity and the risk of vascular complications. In patients presenting with intermittent retinal arterial occlusions, immediate systemic evaluation and a complete hematological workup is crucial, due to the fact that an early diagnosis of PV and prompt treatment can prevent irreversible visual loss and potential life-threatening systemic complications. Since patients with MPN are often undiagnosed until they develop major thrombotic events, ophthalmologists may play an essential role in the diagnosis of the disease. Clinicians must be aware of suspicious ocular thrombotic events without typical predispositions for vascular occlusions (age, smoking, diabetes mellitus, hypertension, glaucoma) and should consider potential polycythemia vera whenever the retinal artery occlusion or branch retinal artery occlusion is encountered.

## Supplementary Information

**Additional file 1. **Detailed pathological-histological examination of the bone marrow biopsy.

**Additional file 2. **Histopathological examination of the bone marrow biopsy. Age correlated slightly hypercellular bone marrow with a marrow cellularity of approx. 50% with rarefied bone trabeculae and homogeneously distributed fat marrow. Haematoxylin-Eosin (HE) stain, 5x magnification. HCX PL FLUOTAR 5x /0.15 (Microscope: LEICA DM 2500, Camera: LEICA EC4, Software: Leica Application Suite LAS4.12).

**Additional file 3. **Histopathological examination of the bone marrow biopsy. Proliferated megakaryopoiesis, the megakaryocytes are in curly clusters and show atypia. Borderline increased erythropoiesis in the background. Morphologically inconspicuous granulopoiesis with maturation. HE stain, 10x magnification. HC PLAN APO 10x/0.40 (Microscope: LEICA DM 2500, Camera: LEICA EC4, Software: Leica Application Suite LAS4.12).

**Additional file 4. **Histopathological examination of the bone marrow biopsy. Megakaryocytes lying in loose clusters with clear atypia: Predominantly large cell shapes with hyperlobulated nuclei are seen. HE stain, 20x magnification. HC PLAN APO 20x/0.70 (Microscope: LEICA DM 2500, Camera: LEICA EC4, Software: Leica Application Suite LAS4.12).

**Additional file 5. **Bone marrow cytology, smear preparation: Megakaryocytes with predominantly large cell shapes and hyperlobulated nuclei. May Grunwald-Giemsa stain, 10x magnification. HC PLAN APO 20x/0.70 (Microscope: LEICA DM 2500, Camera: LEICA EC4, Software: Leica Application Suite LAS4.12).

## Data Availability

The principal data generated and analyzed during the current study are available at NCBI database, accession number PRJNA823882. https://www.ncbi.nlm.nih.gov/bioproject/823882

## Abbreviations

ddPCR: Digital Droplet PCR
JAK2: Janus Kinase 2
HE: Haematoxylin–eosin stain
MPN: Myeloproliferative neoplasm
NGS: Next-Generation-Sequencing
OD: Right eye (oculus dexter)
OS: Left eye (oculus sinister)
PV: Polycythemia vera
SD-OCT: Spectral-domain optical coherence tomography
