# Supplementary material for: Intermittent retinal artery occlusions as the first clinical manifestation of polycythemia vera: a case report
Source: BMC Ophthalmol. 2022 May 15;22:221. doi: 10.1186/s12886-022-02423-w (PMC9107652; doi:10.1186/s12886-022-02423-w)
Supplement: Supplementary file 1 — Additional file 1. Detailed pathological-histological examination of the bone marrow biopsy. [file 12886_2022_2423_MOESM1_ESM.docx]

**Pathological-histological examination of the Bone Marrow**

**Microscopic findings:**

The bone marrow biopsy cylinder is 2 cm in length. It was taken in perfect condition and is representative. We have a case of Spongiosa with an inclusion of 18 assessable marrow spaces with rarified bone trabeculae. Within the medullary canals, there is a largely normocellular bone marrow with a marrow cellularity of approx. 50 % and homogeneously distributed fat marrow. Furthermore, cell elements of a trilinear maturing hematopoiesis are found. In the foreground there is the increased megakaryopoiesis, which also has atypical formations with large cell shapes and hyperlobulated cell nuclei. The present granulopoiesis and erythropoiesis are morphologically inconspicuous. The supplementary immunohistochemical work-up shows a loose infiltration of localized CD20 positive B lymphocytes and CD5 positive T lymphocytes without the formation of lymphocytic aggregates. The reticulin fibre staining shows an inconspicuous fibre architecture without fibre proliferation, corresponding to marrow fibrosis grade 0. No significantly increased storage iron can be detected in the iron staining. In summary, the bone marrow biopsy cylinder shows the picture of a myeloproliferative neoplasia (MPN), primarily of the polycythaemia type. Histomorphological findings are consistent with bone marrow cytology and immunophenotyping.

Assessment:

The findings conclude to a case of age correlated normocellular bone marrow with proliferated atypical megakaryopoiesis, grade 0 marrow fibrosis, histomorphological picture of myeloproliferative neopasia (MPN) of polycythaemia vera (PV) type.

**Bone marrow cytology assessment:**

Prescence of cell-rich bone marrow with increased and atypical megakaryopoiesis. Granulopoiesis (55%) is at the lower norm and is linear. Erythropoiesis is increased at 38% with largely normal maturation. Lymphocytes (5%), plasma cells (0.5%) and blasts (0.5%) are quantitatively normal. Morphologically compatible with an MPN. Due to the polyglobulia (current erythrocytes 5.81 mill./µL, Hk: 51.0%, Hb: 15.7 g/dl) primarily corresponding to a PV.

**Bone marrow immunophenotyping - interpretation:**

No extension of the findings due to the flow cytology.
