# Supplementary figures and images for: Intermittent retinal artery occlusions as the first clinical manifestation of polycythemia vera: a case report
Source: BMC Ophthalmol. 2022 May 15;22:221. doi: 10.1186/s12886-022-02423-w (PMC9107652; doi:10.1186/s12886-022-02423-w)

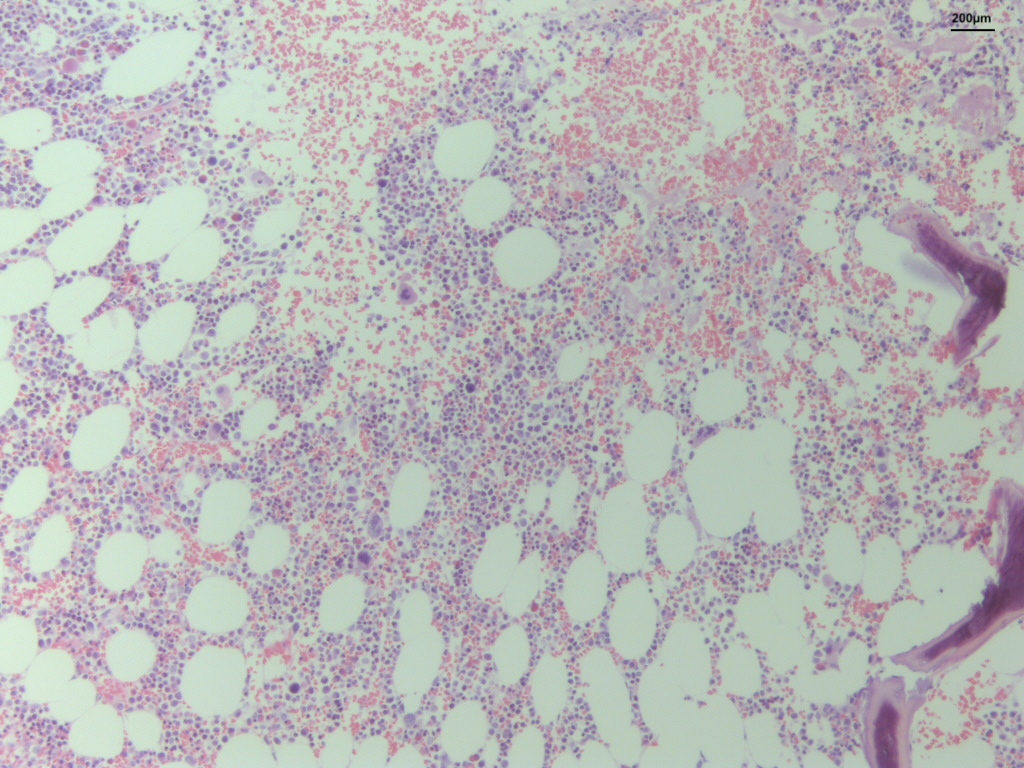

Supplement: Supplementary file 2 — Additional file 2. Histopathological examination of the bone marrow biopsy. Age correlated slightly hypercellular bone marrow with a marrow cellularity of approx. 50% with rarefied bone trabeculae and homogeneously distributed fat marrow. Haematoxylin-Eosin (HE) stain, 5x magnification. HCX PL FLUOTAR 5x /0.15 (Microscope: LEICA DM 2500, Camera: LEICA EC4, Software: Leica Application Suite LAS4.12). [file 12886_2022_2423_MOESM2_ESM.jpg]

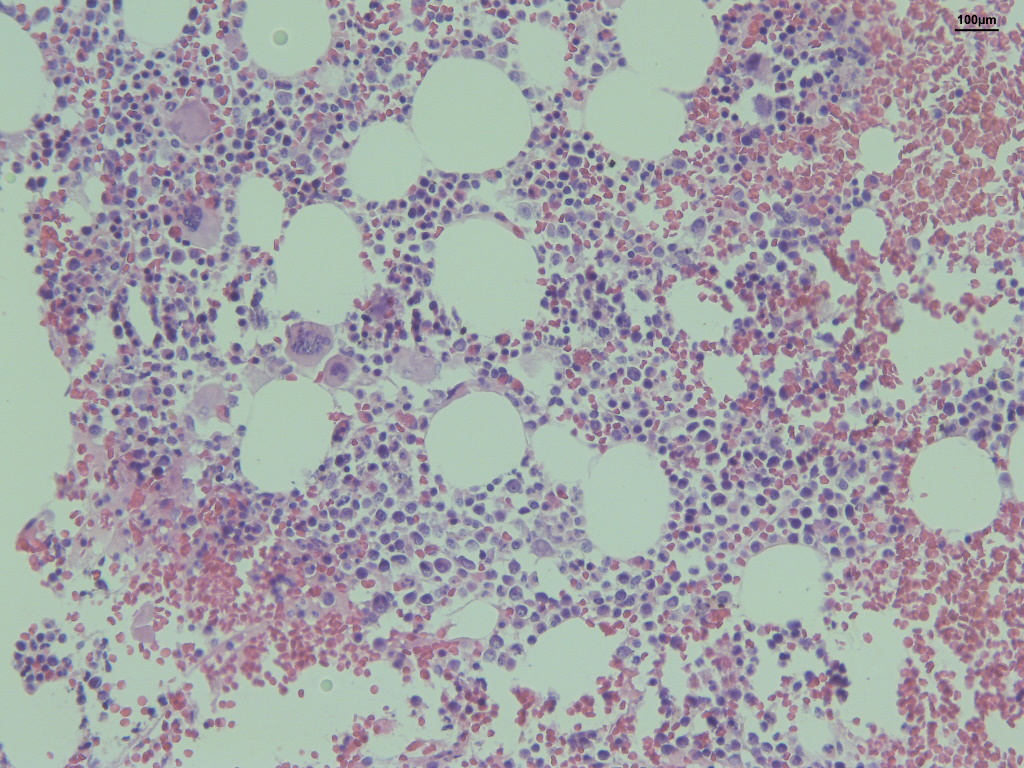

Supplement: Supplementary file 3 — Additional file 3. Histopathological examination of the bone marrow biopsy. Proliferated megakaryopoiesis, the megakaryocytes are in curly clusters and show atypia. Borderline increased erythropoiesis in the background. Morphologically inconspicuous granulopoiesis with maturation. HE stain, 10x magnification. HC PLAN APO 10x/0.40 (Microscope: LEICA DM 2500, Camera: LEICA EC4, Software: Leica Application Suite LAS4.12). [file 12886_2022_2423_MOESM3_ESM.jpg]

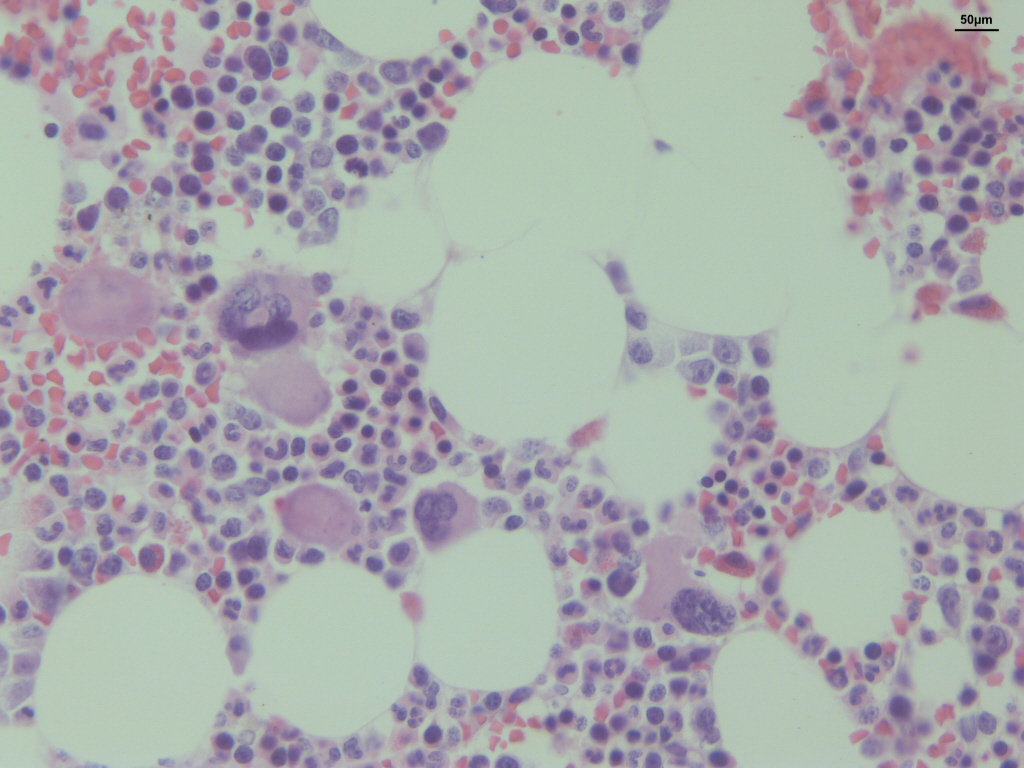

Supplement: Supplementary file 4 — Additional file 4. Histopathological examination of the bone marrow biopsy. Megakaryocytes lying in loose clusters with clear atypia: Predominantly large cell shapes with hyperlobulated nuclei are seen. HE stain, 20x magnification. HC PLAN APO 20x/0.70 (Microscope: LEICA DM 2500, Camera: LEICA EC4, Software: Leica Application Suite LAS4.12). [file 12886_2022_2423_MOESM4_ESM.jpg]

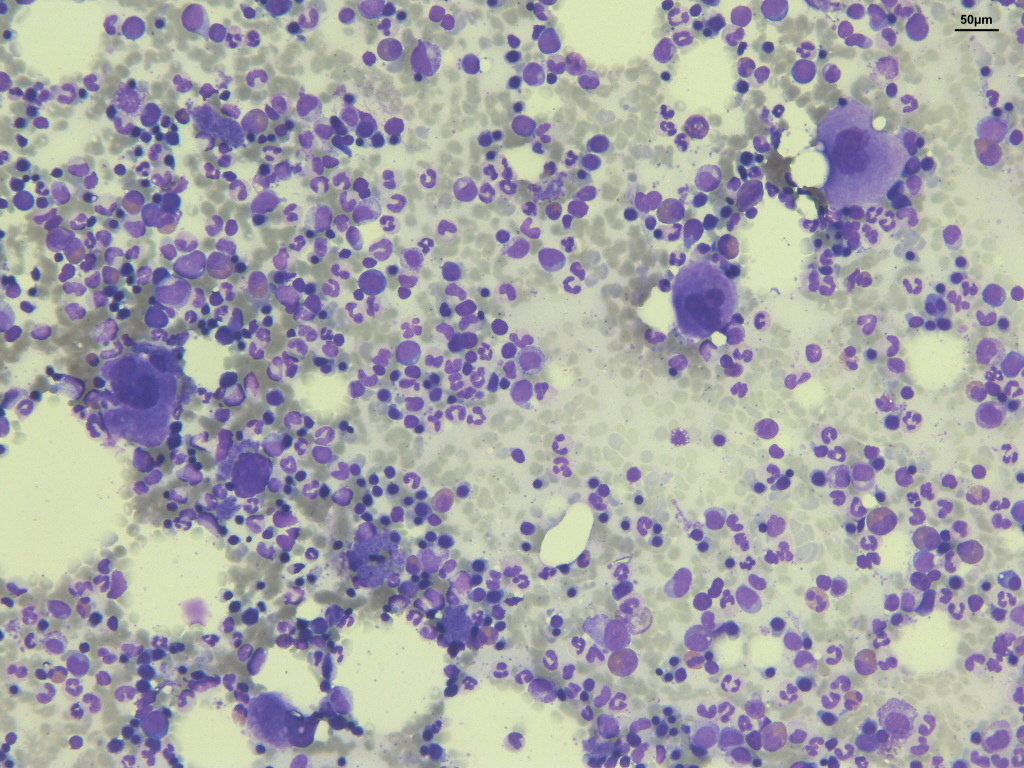

Supplement: Supplementary file 5 — Additional file 5. Bone marrow cytology, smear preparation: Megakaryocytes with predominantly large cell shapes and hyperlobulated nuclei. May Grunwald-Giemsa stain, 10x magnification. HC PLAN APO 20x/0.70 (Microscope: LEICA DM 2500, Camera: LEICA EC4, Software: Leica Application Suite LAS4.12). [file 12886_2022_2423_MOESM5_ESM.jpg]
